# Supplementary material for: Deep-profiling of phospholipidome via rapid orthogonal separations and isomer-resolved mass spectrometry
Source: Nat Commun. 2023 Jul 17;14:4263. doi: 10.1038/s41467-023-40046-x (PMC10352238; doi:10.1038/s41467-023-40046-x)
Supplement: Supplementary file 1 — Supplementary Information [file 41467_2023_40046_MOESM1_ESM.pdf]

Supplementary Information for

Deep-profiling of phospholipidome via rapid orthogonal separations and isomer-resolved mass spectrometry

Xia *et al.*

Includes:

- Supplementary Figures 1-24
- Supplementary Tables 1-2
- Supplementary Notes 1-2
- Supplementary Methods
- Supplementary References

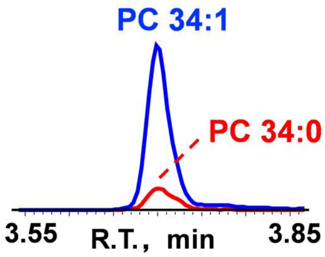

Supplementary Fig. 1. Extracted ion chromatogram (EIC) of PC 34:0 (red trace) and PC 34:1 (blue trace) via HILIC separation.

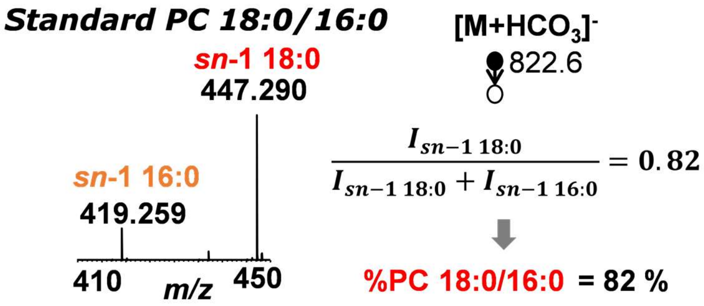

Supplementary Fig. 2. MS^2 CID of $[M + HCO_3]^-$ of PC 18:0/16:0 (standard).

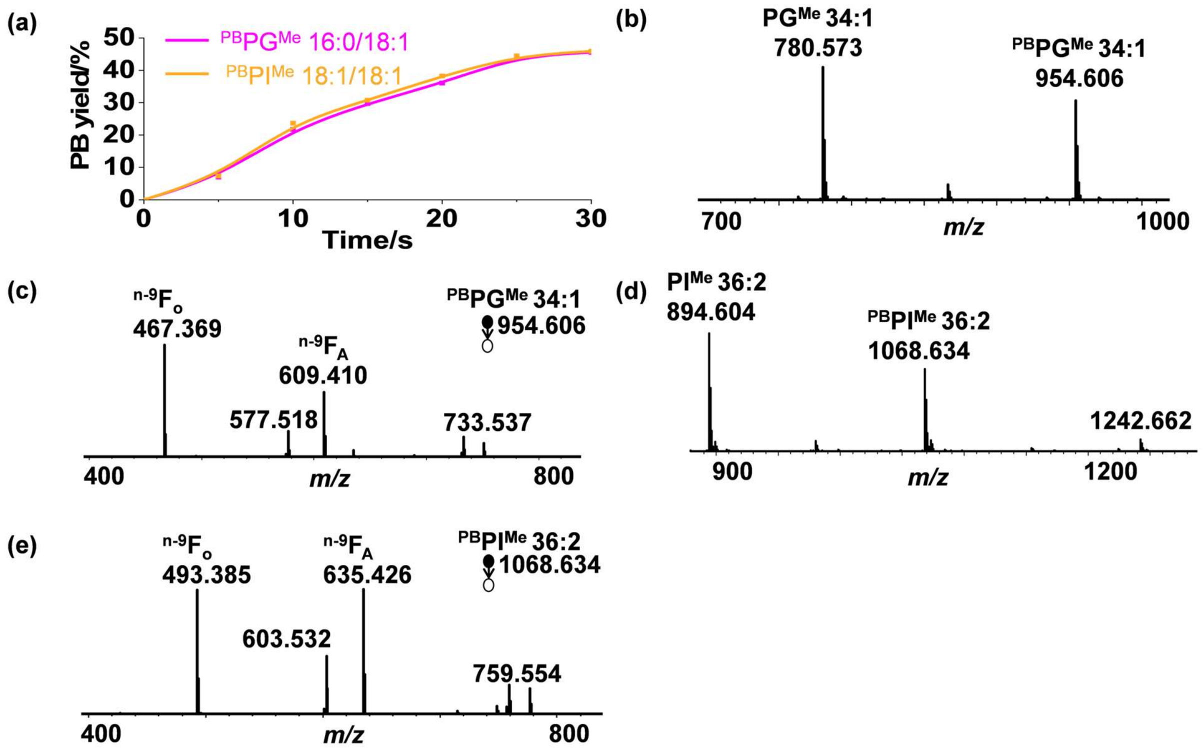

Supplementary Fig. 3. (a) The %PB yield of the triFAP derivatization of methylated PG (PG^{Me}) 16:0/18:1(9Z) and methylated PI (PI^{Me}) 18:1(9Z)/18:1(9Z) as a function of reaction time, respectively. (b) MS^1 spectrum of the triFAP PB reaction of PG^{Me} 16:0/18:1 (10 μM) performed using a flow microreactor after 20s' UV irradiation. (c) MS^2 CID spectrum of the triFAP PB products of PG^{Me} 16:0/18:1 ($[^{PB}PG^{Me} + NH_4]^+$, *m*/z 954.6). (d) MS^1 spectrum of the triFAP PB reaction of PI^{Me} 18:1/18:1 (10 μM) performed using a flow microreactor under 20s' UV irradiation. (e) MS^2 CID spectrum of the triFAP PB products of PI^{Me} 18:1/18:1 ($[^{PB}PI^{Me} + NH_4]^+$, *m*/z 1068.6).

Note: We paired phosphate methylation with triFAP PB reaction for sensitive and structurally informative analysis of PG and PI down to C=C location. Almost quantitative conversion was achieved for phosphate methylation within 20 min. The PB conversion was obtained at 30-40% for methylated PG (PG^{Me}) and PI (PI^{Me}) after 20 s' reaction. The C=C diagnostic ions were detected as the most abundant fragment ions in the PB-MS/MS spectra of PG^{Me} and PI^{Me}.

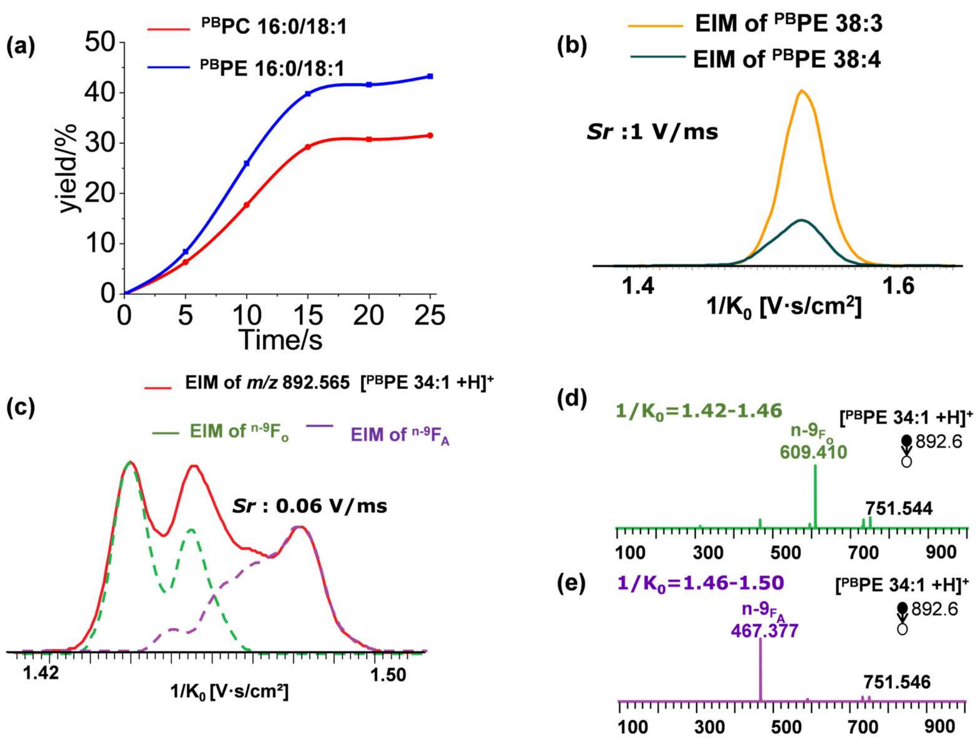

Supplementary Fig. 4. (a) %PB yield of triFAP derivatization of PC 16:0/18:1(9Z) and PE 16:0/18:1(9Z) as a function of reaction time, respectively. (b) EIMs of triFAP-modified PE 38:3 (orange trace) and triFAP-modified PE 38:4 (dark green trace). (c) EIMs of $[^{PB}PE\ 16:0/18:1+H]^+$ (*m/z* 892.57, red trace), C=C diagnostic fragment ions $^{n\text{-}9}F_O$ (*m/z* 609.41, green dots), and $^{n\text{-}9}F_A$ (*m/z* 467.38, purple dots). Extracted MS/MS spectra of $[^{PB}PE\ 16:0/18:1+H]^+$ from different $1/K_0$ ranges: (d) 1.42-1.46 and (e) 1.46−1.50.

Note: The PB products are always a mixture of several isomers. The two regio-isomers, each lead to form $^{n\text{-}9}F_O$ and $^{n\text{-}9}F_A$ upon CID, are partially resolved by TIMS. MS^2 CID of the later eluted doublets at $1/K_0$ 1.42−1.46 produces C=C diagnostic ions at *m/z* 609.41, corresponding to $^{n\text{-}9}F_O$. The mobility doublets arise from partially separated *cis*- and *trans*-stereo isomers of the oxetane ring. MS^2 CID of the early eluted peak at $1/K_0$ 1.46−1.50 produces a prominent aldehyde fragment ($^{n\text{-}9}F_O$) at *m/z* 467.38.

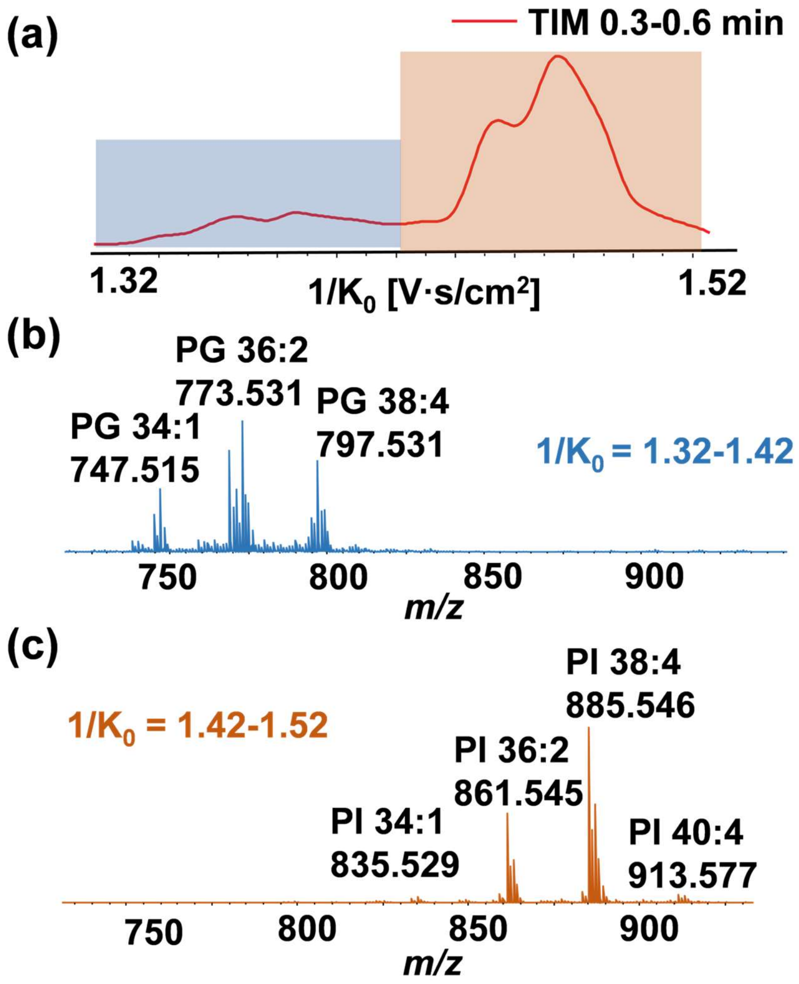

Supplementary Fig. 5. (a) Total ion mobilograms eluted at 0.3-0.6 min. Extracted MS[1] spectra from different $1/K_0$ ranges: (b) 1.32-1.42 and (c) 1.42-1.52.

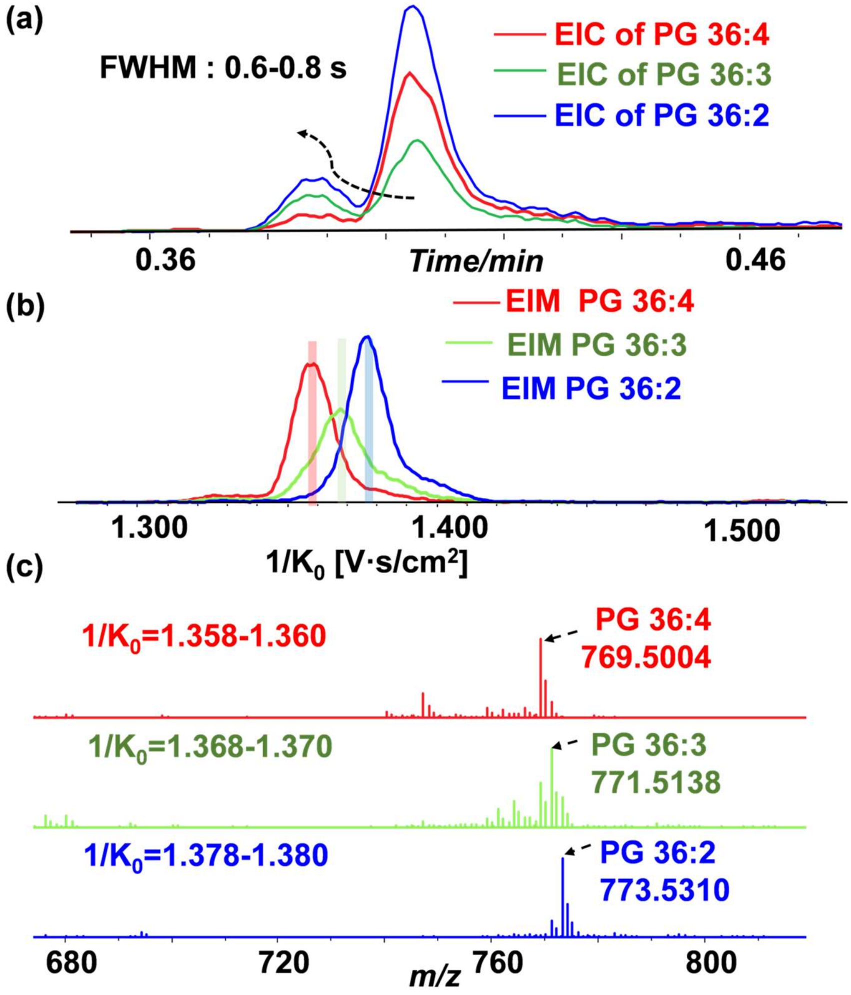

Supplementary Fig. 6. (a) EICs of PG 36:4 (red trace), PG 36:3 (green trace), and PG 36:2 (blue trace). (b) EIMs of PG 36:4 (red trace), PG 36:3 (green trace) and PG 36:2(blue trace).(c) Extracted MS^1 spectra from different $1/K_0$ ranges:1.358-1.360, 1.368-1.370, and 1.378-1.380.

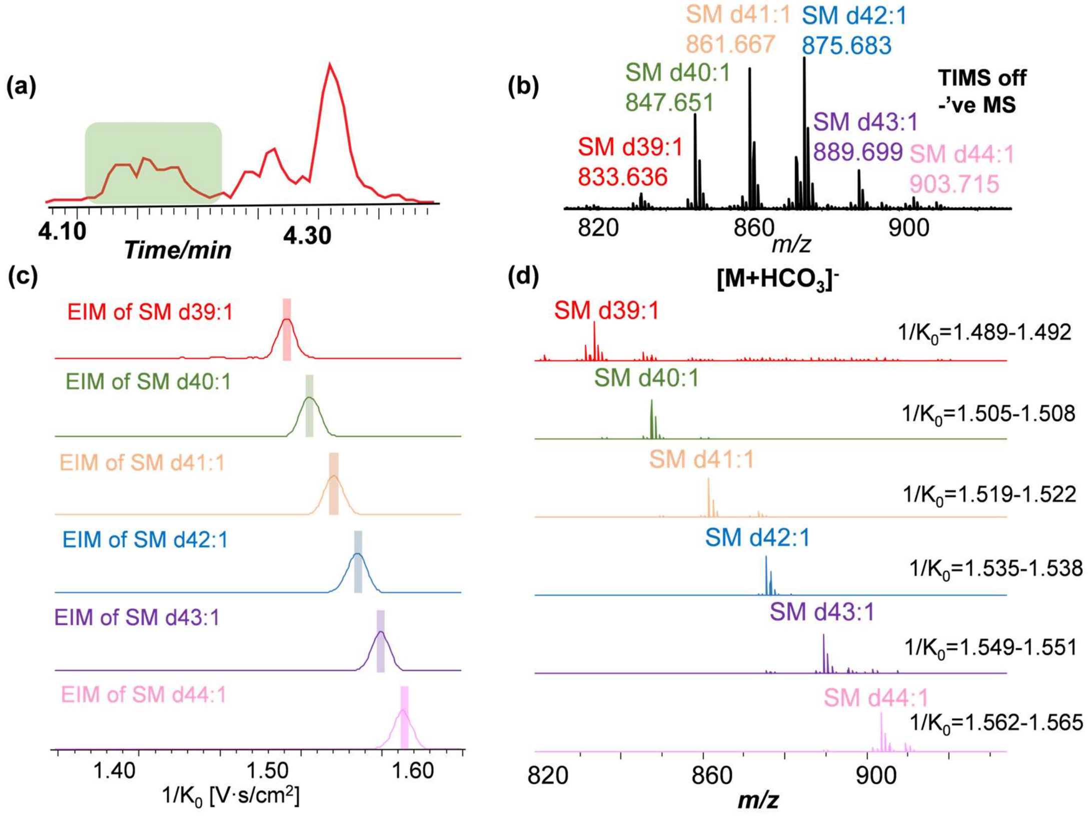

Supplementary Fig. 7. (a). Total ion chromatogram eluted at 4.1-4.4 min and (b) corresponding MS^1 spectrum.(c) EIMs of SM d39:1, SM d40:1, SM d41:1, SM d42:1, SM d43:1, and SM d44:1. (d) Extracted MS^1 spectra from different $1/K_0$ ranges: 1.489-1.492, 1.505-1.508, 1.519-1.522, 1.535-1.538, 1.549-1.551, and 1.562-1.565.

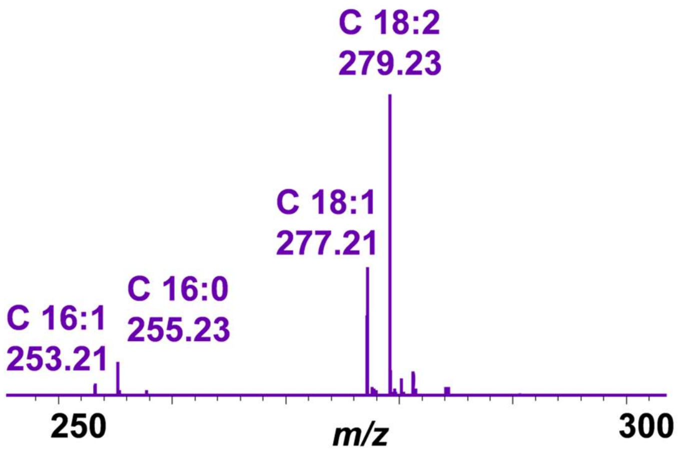

Supplementary Fig. 8. MS^2 CID of *m/z* 712.5 in bovine liver polar lipid extract without TIMS separation. Only the *m/z* region of the fatty acyl fragment ions is shown.

Note: Without TIMS separation, PE 34:3 (*m/z* 712.4923) and PE O-35:3 (*m/z* 712.5287) are co-isolated during MS/MS, which leads to inconclusive identification for chain composition. We may misidentify PE O-35:3 as a mixture of PE O-17:1_18:2, PE O-17:0_18:3, PE O-19:3_16:0 and PE O-19:2_16:1, and identify PE 34:3 as a mixture of PE 16:0_18:3 and PE 16:1_18:2.

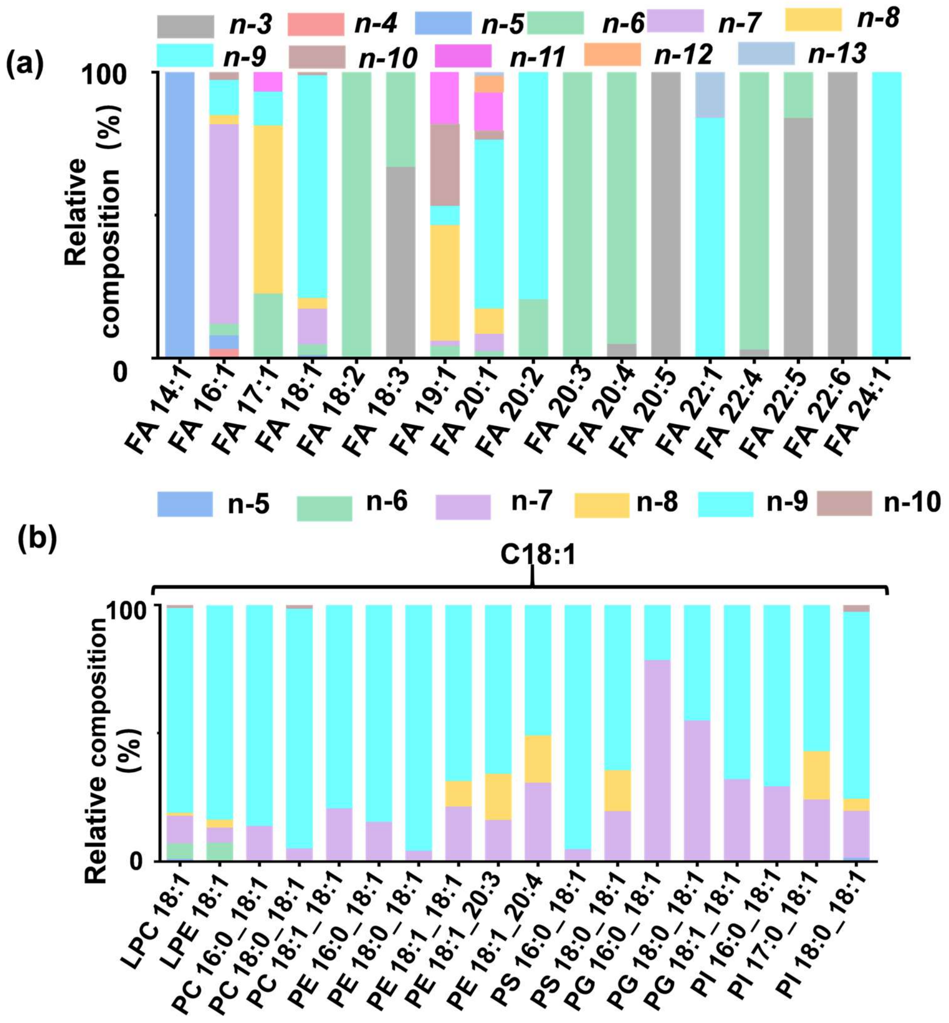

Supplementary Fig. 9. (a) Relative compositions (%) of C=C location isomers in 17 groups of total FAs in bovine liver. (b) Relative compositions (%) of 18 groups of C=C location isomers for LPC, LPE, PC, PE, PS, PG, and PI containing C18:1 acyl chain.

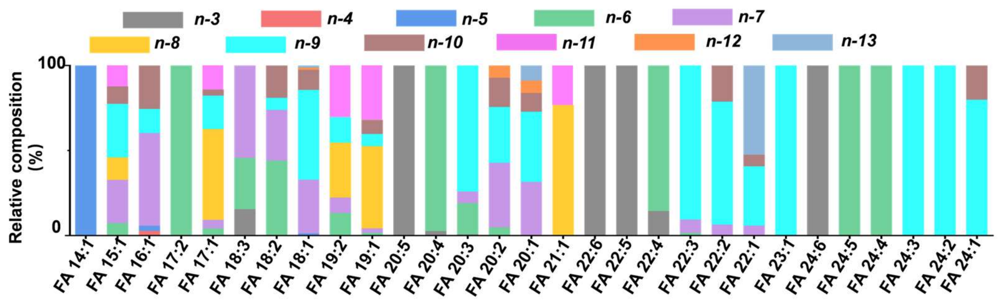

Supplementary Fig. 10. Relative compositions (%) of C=C location isomers in 29 groups of FAs in RAW 264.7 macrophages lipid extracts (N=6).

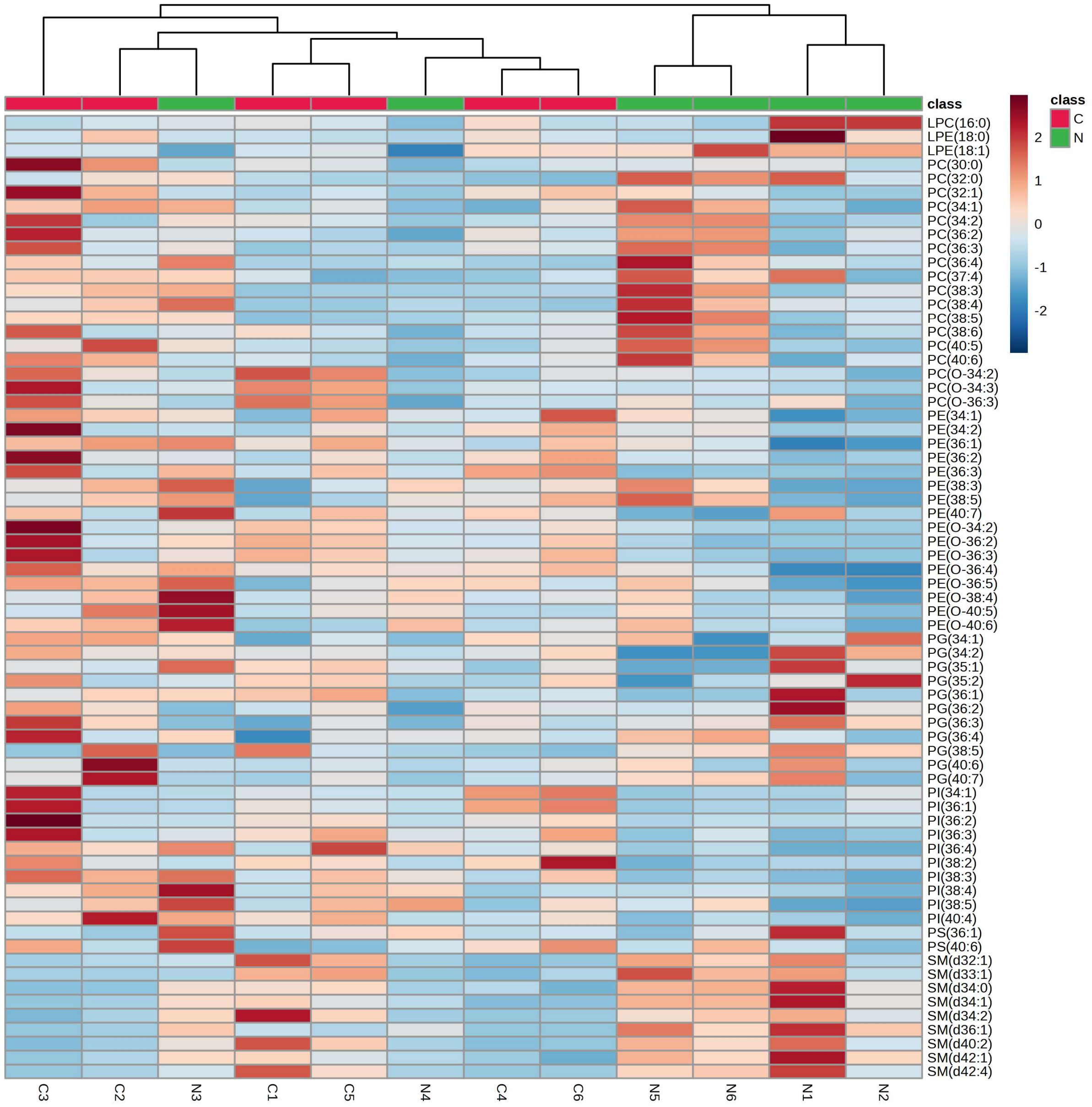

Supplementary Fig. 11. Heatmap of relative ion intensities of glycerophospholipids (GPLs) and sphingomyelins (SMs) found in normal (N1 to N6) and bladder cancerous (C1 to C6) tissue samples. Colors represent different relative intensities as indicated by the color bar.

Supplementary Table 1. Comparison of the changes in full width at half maximum (FWHM) of the mobility peak of lipids before and after TriFAP PB derivatization

	FWHM 1/k_0 $[M+H]^+$	FWHM 1/k_0 $[M+triFAP+H]^+$
PE 34:1	0.022	0.036
PE 36:1	0.023	0.038
PE 36:2	0.022	0.036
PE 38:3	0.022	0.036
PE 36:2	0.021	0.035

Supplementary Table 2. Comparison of experimental collision cross-section (CCS) values obtained in this work with those reported in the existing literature

	this work	Lerner et al, Nat. Commun. 2023, 14, 937[1]	Leaptrot et al, Nat. Commun. 2019, 10, 985[2]
Instrument platform	Trapped ion mobility	Trapped ion mobility	Drift Tube ion mobility
LPE 16:0, CCS $[M-H]^-$(Å^2)	209.5	210.5	No report
LPE 18:0, CCS $[M-H]^-$(Å^2)	216.9	218.1	No report
PE 34:1, CCS $[M-H]^-$(Å^2)	268.6	268.5	266.9
PE 36:2, CCS $[M-H]^-$(Å^2)	273.8	273.5	272.1
PE 38:4 , CCS $[M-H]^-$(Å^2)	278.1	277.4	276.0
PE O-36:2 , CCS $[M-H]^-$(Å^2)	274.3	274.3	269.0
PE O-38:4 , CCS $[M-H]^-$(Å^2)	278.2	277.6	No report
PI 34:1, CCS $[M-H]^-$(Å^2)	290.5	291.3	286.4
PI 36:2, CCS $[M-H]^-$(Å^2)	295.5	295.4	292.0
PI 38:4 , CCS $[M-H]^-$(Å^2)	300.0	299.1	296.4
PG 34:1, CCS $[M-H]^-$(Å^2)	276.0	275.5	273.1
PG 36:2, CCS $[M-H]^-$(Å^2)	281.3	282.1	279.5
PS 36:2, CCS $[M-H]^-$(Å^2)	283.7	283.5	281.8
PS 38:4, CCS $[M-H]^-$(Å^2)	287.6	287.5	285.3

Supplementary Note 1. LipidNovelist Tutorial

Introduction

LipidNovelist is a python-based software tool designed for the annotation of lipid structures from liquid chromatography-mass spectrometry (LC-MS) data. . The program allows for annotation at the lipid subclass level, chain composition level, C=C location level, and *sn* isomer level. The graphical user interface (GUI) provides details of data information, input parameters, annotation results, and related tandem mass spectra (MS/MS) visualization. The current version of LipidNovelist, example data and tutorial videos for ease of use is available at https://doi.org/10.6084/m9.figshare.22297771

Supplementary Fig. 12 displays a screenshot of LipidNovelist graphical user interface (GUI), which can be roughly divided into five sections. Firstly, the 'Parameter Column' section (Brown) is used for data import and parameter selection. Secondly, the 'Raw data list' section (Blue) provides visualization of raw data, displaying the retention time of each scan. Thirdly, the top middle section of the GUI shows the MS/MS spectra or TIC spectrum, which can be found in the 'Spectrum Visualization' section (Green). Fourthly, the button middle section of the GUI displays lipid identification results. It contains two tables, one for showing results of lipids at subclass, chain or sn position level, and the other for showing results of lipids at C=C location level (Yellow). Lastly, LipidNovelist offers data preprocessing capability to form pseudo-precursor ion scan (PIS)/neutral loss scan (NLS) spectra. The detailed information of the results is presented in the right column (Black).

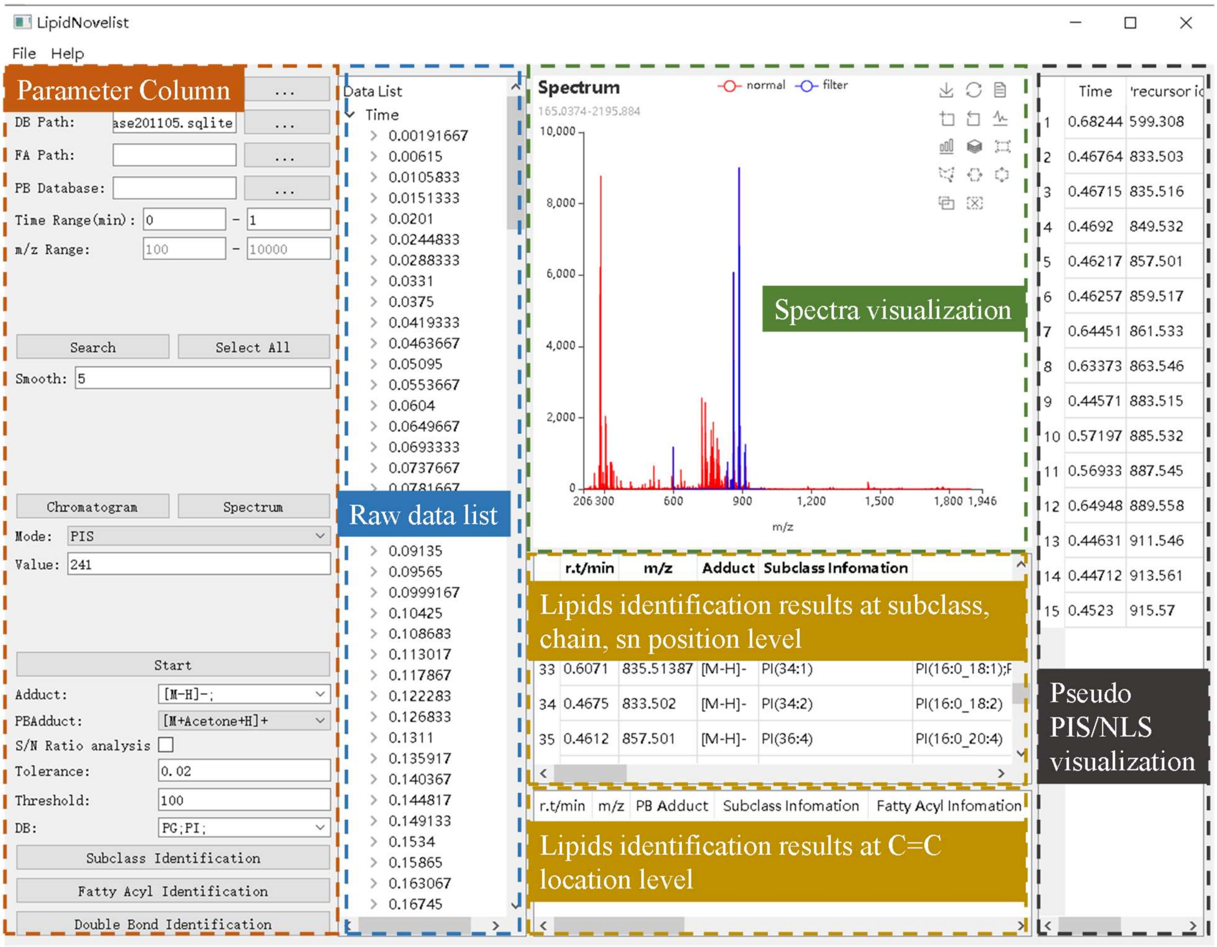

Supplementary Fig. 12. Screenshot of LipidNovelist.

Chapter 1 Data format conversion

LipidNovelist has the capability to read .ascii files that can be exported by the DataAnalysis tool in Bruker. To use data collected from other mass spectrometry (MS) instruments, the data must be converted to .mzML files using Proteowizard's MSConvert tool, and then converted to .ascii files using a custom-built converter, which can be downloaded at https://doi.org/10.6084/m9.figshare.22297771

The converter's graphical user interface (GUI) is depicted in Supplementary Fig. 13. The user can import data in .mzML format by clicking the "Read" button. The directory where the exported .ascii file will be saved can be changed by clicking the "Save Dir" button. The file name

and directory can also be modified by editing the relevant lines. After the changes have been made, the "Save" button can be clicked, and the data will be exported in .ascii format.

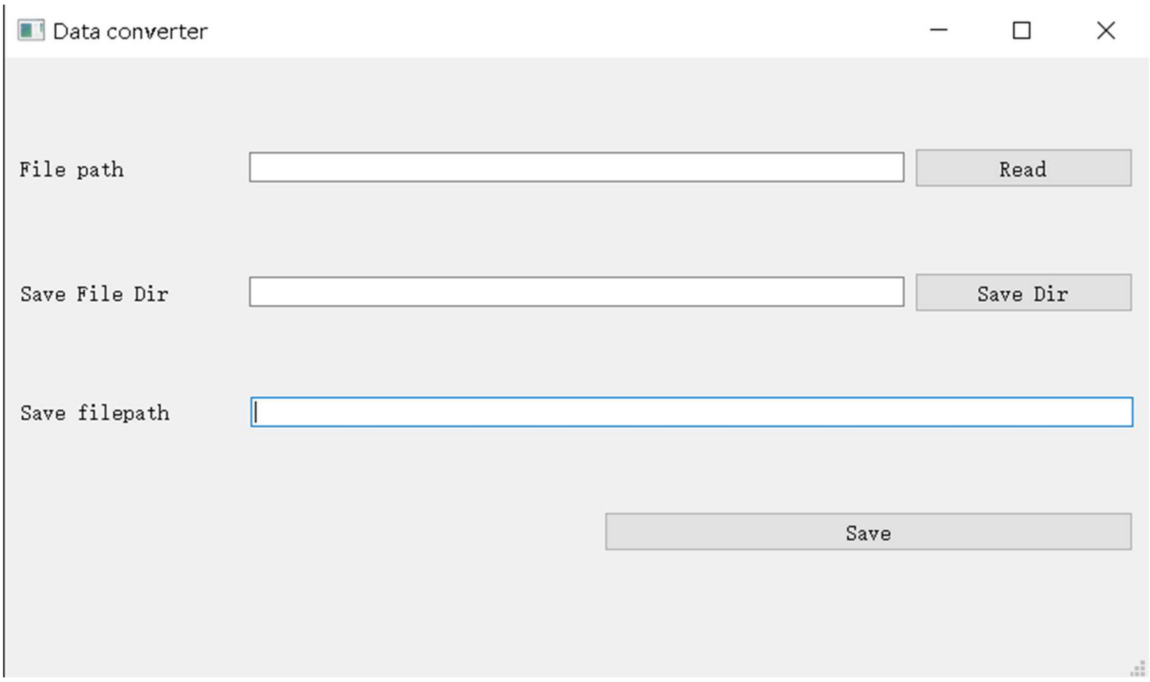

Supplementary Fig. 13. GUI of the data format converter.

Chapter 2 Data import

The present chapter describes the process of importing data and carrying out basic preprocessing steps in LipidNovelist. In the top left corner of the graphical user interface (GUI), data can be imported and basic preprocessing can be performed (Supplementary Fig. 14). By clicking on the button located on the same line as "Record Path," users can select and read data in the .ascii format. At multiple level of lipid annotation, a .sqlite format database sourced from LIPID MAPS is required. A pre-built .sqlite file can be found in the 'database' subfolder of the main folder of LipidNovelist. Further details on the database are available in Supplementary Fig. 15. When analyzing data from the Paterno-Buchi (PB) reaction coupled with LC-MS/MS experiment, two additional files are required for lipid C=C location annotation. The formats of these files are introduced in the subsequent chapters. Chromatography can be visualized, and the demonstration can be found in the "Show chromotagraphy.mp4" video tutorial.

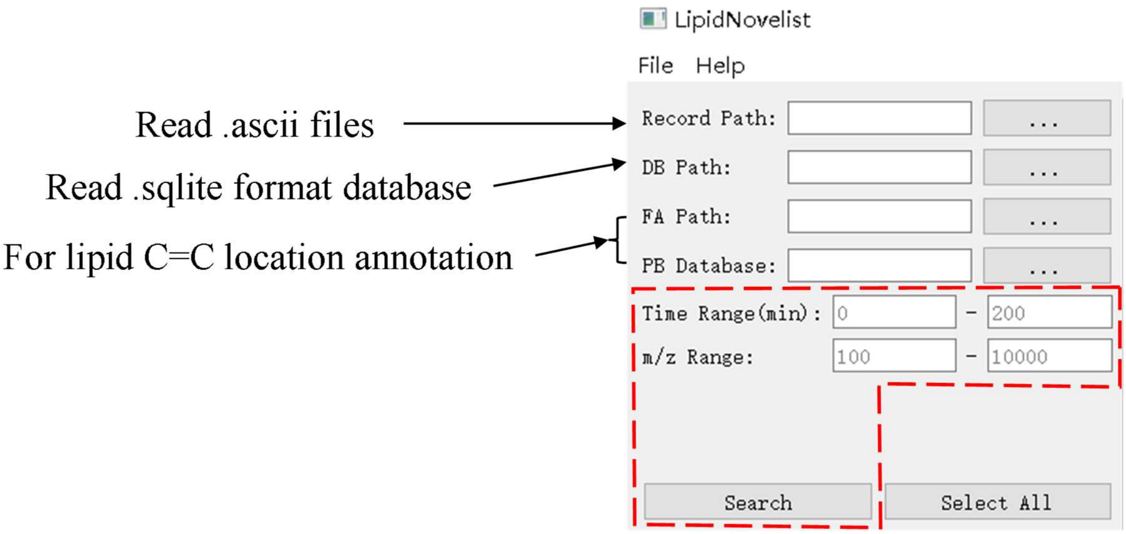

Supplementary Fig. 14. Data import and basic preprocessing

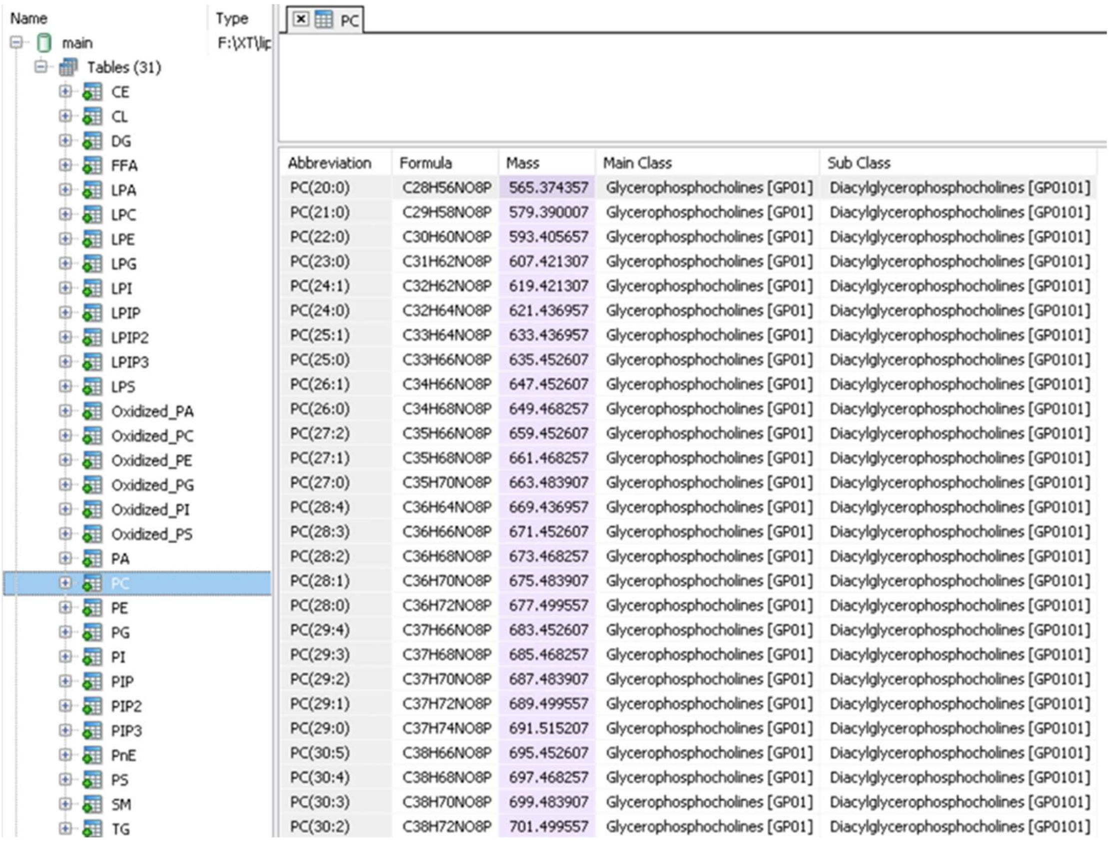

Abbreviation	Formula	Mass	Main Class	Sub Class
PC(20:0)	C28H56NO8P	565.374357	Glycerophosphocholines [GP01]	Diacylglycerophosphocholines [GP0101]
PC(21:0)	C29H58NO8P	579.390007	Glycerophosphocholines [GP01]	Diacylglycerophosphocholines [GP0101]
PC(22:0)	C30H60NO8P	593.405657	Glycerophosphocholines [GP01]	Diacylglycerophosphocholines [GP0101]
PC(23:0)	C31H62NO8P	607.421307	Glycerophosphocholines [GP01]	Diacylglycerophosphocholines [GP0101]
PC(24:1)	C32H62NO8P	619.421307	Glycerophosphocholines [GP01]	Diacylglycerophosphocholines [GP0101]
PC(24:0)	C32H64NO8P	621.436957	Glycerophosphocholines [GP01]	Diacylglycerophosphocholines [GP0101]
PC(25:1)	C33H64NO8P	633.436957	Glycerophosphocholines [GP01]	Diacylglycerophosphocholines [GP0101]
PC(25:0)	C33H66NO8P	635.452607	Glycerophosphocholines [GP01]	Diacylglycerophosphocholines [GP0101]
PC(26:1)	C34H66NO8P	647.452607	Glycerophosphocholines [GP01]	Diacylglycerophosphocholines [GP0101]
PC(26:0)	C34H68NO8P	649.468257	Glycerophosphocholines [GP01]	Diacylglycerophosphocholines [GP0101]
PC(27:2)	C35H66NO8P	659.452607	Glycerophosphocholines [GP01]	Diacylglycerophosphocholines [GP0101]
PC(27:1)	C35H68NO8P	661.468257	Glycerophosphocholines [GP01]	Diacylglycerophosphocholines [GP0101]
PC(27:0)	C35H70NO8P	663.483907	Glycerophosphocholines [GP01]	Diacylglycerophosphocholines [GP0101]
PC(28:4)	C36H64NO8P	669.436957	Glycerophosphocholines [GP01]	Diacylglycerophosphocholines [GP0101]
PC(28:3)	C36H66NO8P	671.452607	Glycerophosphocholines [GP01]	Diacylglycerophosphocholines [GP0101]
PC(28:2)	C36H68NO8P	673.468257	Glycerophosphocholines [GP01]	Diacylglycerophosphocholines [GP0101]
PC(28:1)	C36H70NO8P	675.483907	Glycerophosphocholines [GP01]	Diacylglycerophosphocholines [GP0101]
PC(28:0)	C36H72NO8P	677.499557	Glycerophosphocholines [GP01]	Diacylglycerophosphocholines [GP0101]
PC(29:4)	C37H66NO8P	683.452607	Glycerophosphocholines [GP01]	Diacylglycerophosphocholines [GP0101]
PC(29:3)	C37H68NO8P	685.468257	Glycerophosphocholines [GP01]	Diacylglycerophosphocholines [GP0101]
PC(29:2)	C37H70NO8P	687.483907	Glycerophosphocholines [GP01]	Diacylglycerophosphocholines [GP0101]
PC(29:1)	C37H72NO8P	689.499557	Glycerophosphocholines [GP01]	Diacylglycerophosphocholines [GP0101]
PC(29:0)	C37H74NO8P	691.515207	Glycerophosphocholines [GP01]	Diacylglycerophosphocholines [GP0101]
PC(30:5)	C38H66NO8P	695.452607	Glycerophosphocholines [GP01]	Diacylglycerophosphocholines [GP0101]
PC(30:4)	C38H68NO8P	697.468257	Glycerophosphocholines [GP01]	Diacylglycerophosphocholines [GP0101]
PC(30:3)	C38H70NO8P	699.483907	Glycerophosphocholines [GP01]	Diacylglycerophosphocholines [GP0101]
PC(30:2)	C38H72NO8P	701.499557	Glycerophosphocholines [GP01]	Diacylglycerophosphocholines [GP0101]

Supplementary Fig. 15. Glycerophosphocholines (PC) in .sqlite database.

In Supplementary Fig. 14, the time range and *m/z* range are optional parameters for further lipid annotation. Once these ranges are filled in, users should click on the "Search" button to constrain the LC-MS/MS spectra within the range. After importing the data, raw data will be listed in the "Data List" column, as shown in Supplementary Fig. 16 . Upon clicking the 'Search' button, data with a limited retention time and *m/z* range will be displayed in the 'Data List' column.

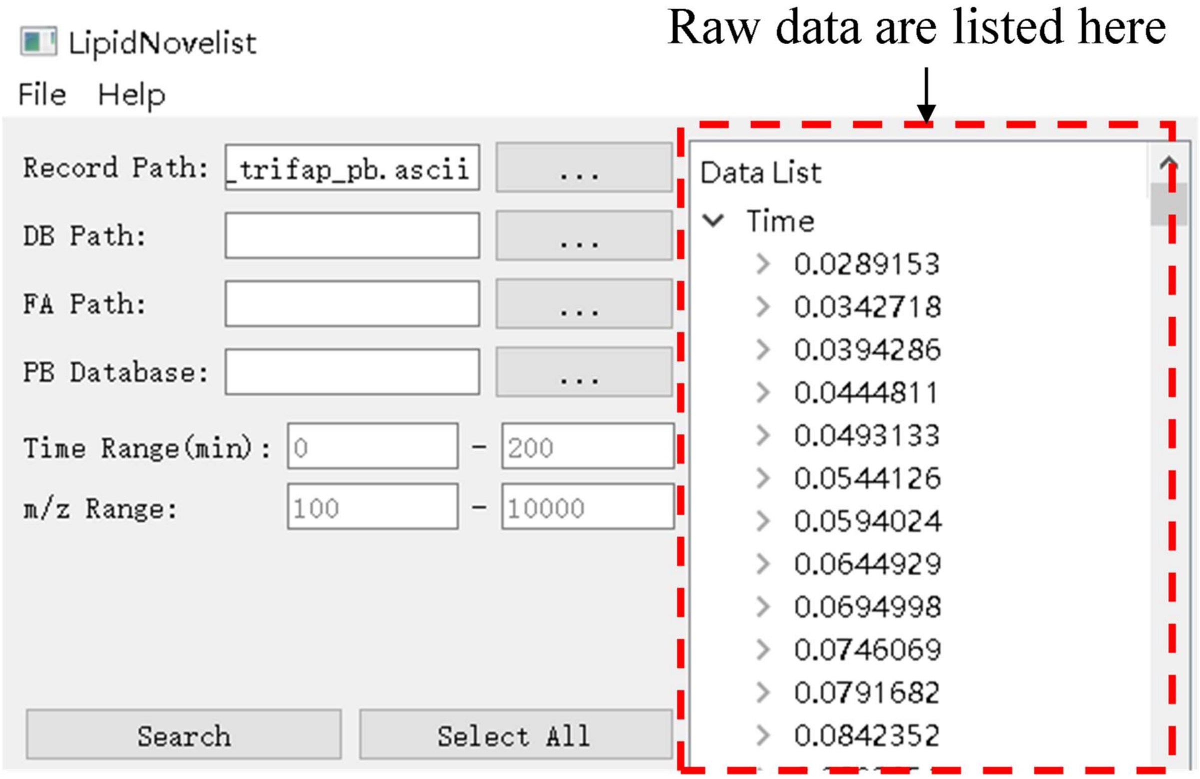

Supplementary Fig. 16. LipidNovelist list raw data in "Data List" column.

Chapter 3 Data preprocessing

This chapter describes the process of forming pseudo precursor ion scan (PIS) or neutral loss scan (NLS) spectra for further lipid identification at the species level. By selecting the PIS/NLS mode and filling in the exact *m/z* or mass weight of neutral loss molecules, LipidNovelist helps users to select specific peaks in MS/MS and form the spectra by taking the *m/z* of precursors as x values and intensities of these specific peaks as y values (Supplementary Fig. 17).

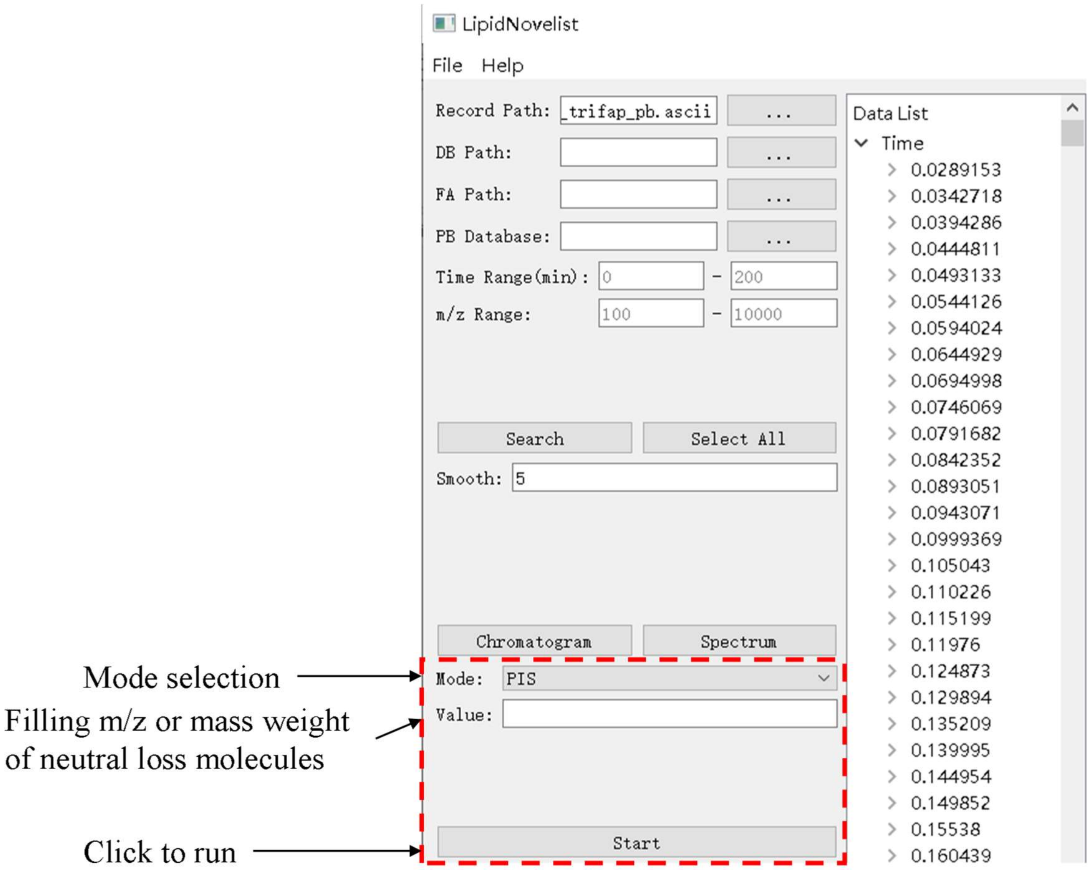

Supplementary Fig. 17. Place to select parameters in Pseudo-PIS/NLS mode

As an example, when selecting PIS at m/z 241, clicking the 'Start' button in Supplementary Fig. 18 will display the retention time and related m/z of the precursor on the right side of the GUI (Supplementary Fig. 18, red). After clicking the 'Spectrum' button in the parameter column, a pseudo-PIS/NLS spectrum will be shown. The red line represents the MS[1] spectrum, and the blue line represents the selected precursors (Supplementary Fig. 18, blue). A demonstration of the pseudo-PIS/NLS process is available in the "Psedeo_PIS_NLS.mp4" video tutorial.

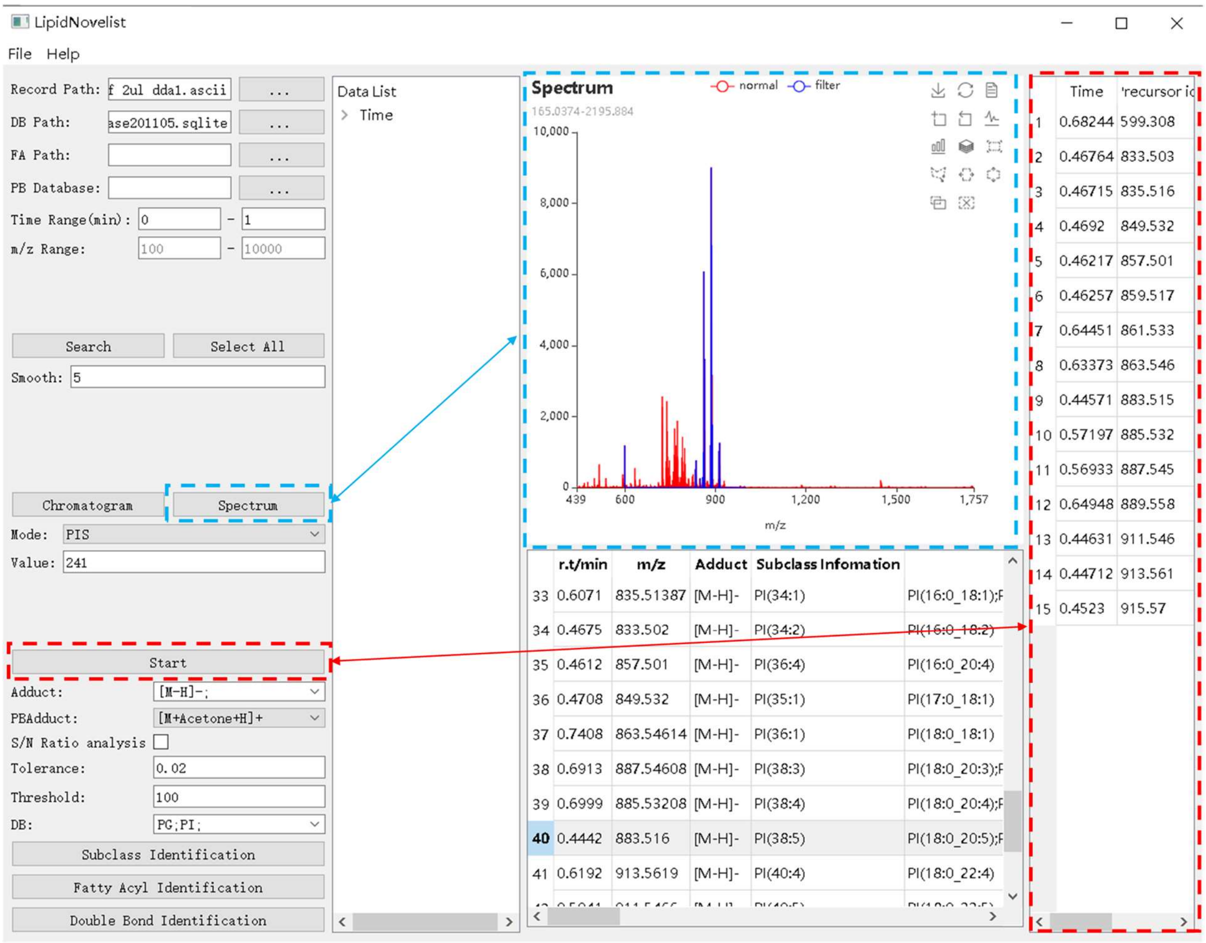

Supplementary Fig. 18. visualization of PIS/NLS results

Chapter 4 Lipid annotation

This chapter provides information on selecting additional parameters required for lipid identification, as shown in Supplementary Fig. 19. Except for parameters specific to PB-MS/MS analysis, other parameters need to be filled in to achieve lipid identification. Apart from parameters unique to PB-MS/MS analysis, these parameters must be filled in to attain successful lipid identification. These parameters comprise:

1.The adduct of lipids;

2.S/N analysis usage to filter noise peaks in MS/MS spectra (optional);

3.mass tolerance to match peaks with the theoretical *m/z* of precursors;

4.absolute intensity threshold to filter noise peaks;

5.the lipid species to be analyzed (multi-select)

After filling in these parameters, spectra can be analyzed to identify lipids at the sum composition level (by clicking 'Subclass Identification'), chain composition level (by clicking 'Fatty Acyl Identification'), and *sn* position level (by clicking 'Fatty Acyl Identification,' specific to PCs when HCO_3^- is used as the adduct).

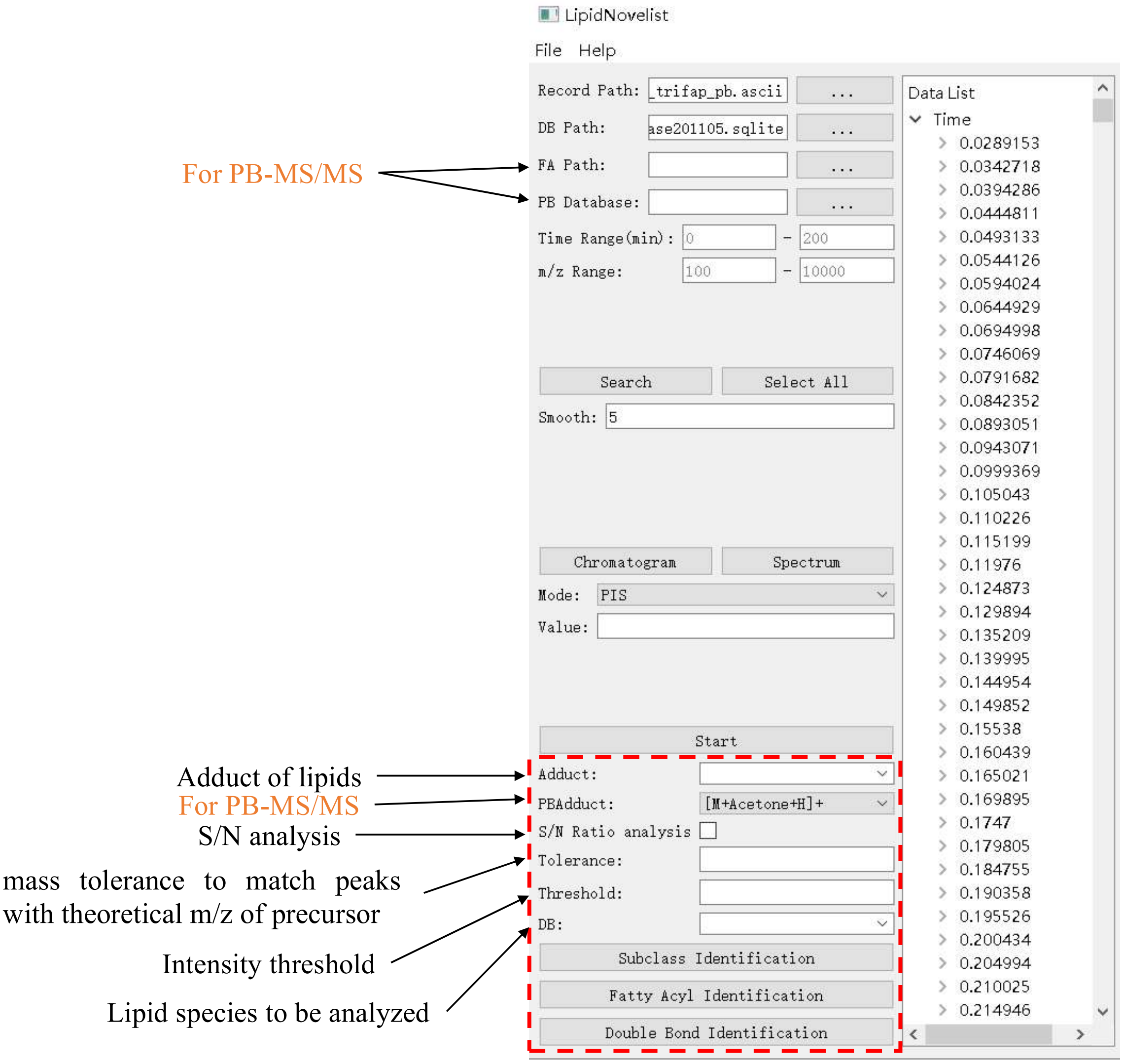

Supplementary Fig. 19. Parameters for lipid annotation

Results of lipid annotation at the subclass level, chain composition level, and sn position

level are displayed in the mid-table (Supplementary Fig. 20 red). The table includes columns containing retention time, m/z, adduct, subclass information, fatty acyl chain information, and sn information. Results of identification are shown in rows, where each row contains lipids with the same subclass information. A demonstration of glycerophospholipid annotation at the chain composition level and *sn* position level can be found in the "chain and sn analysis.mp4" video tutorial.

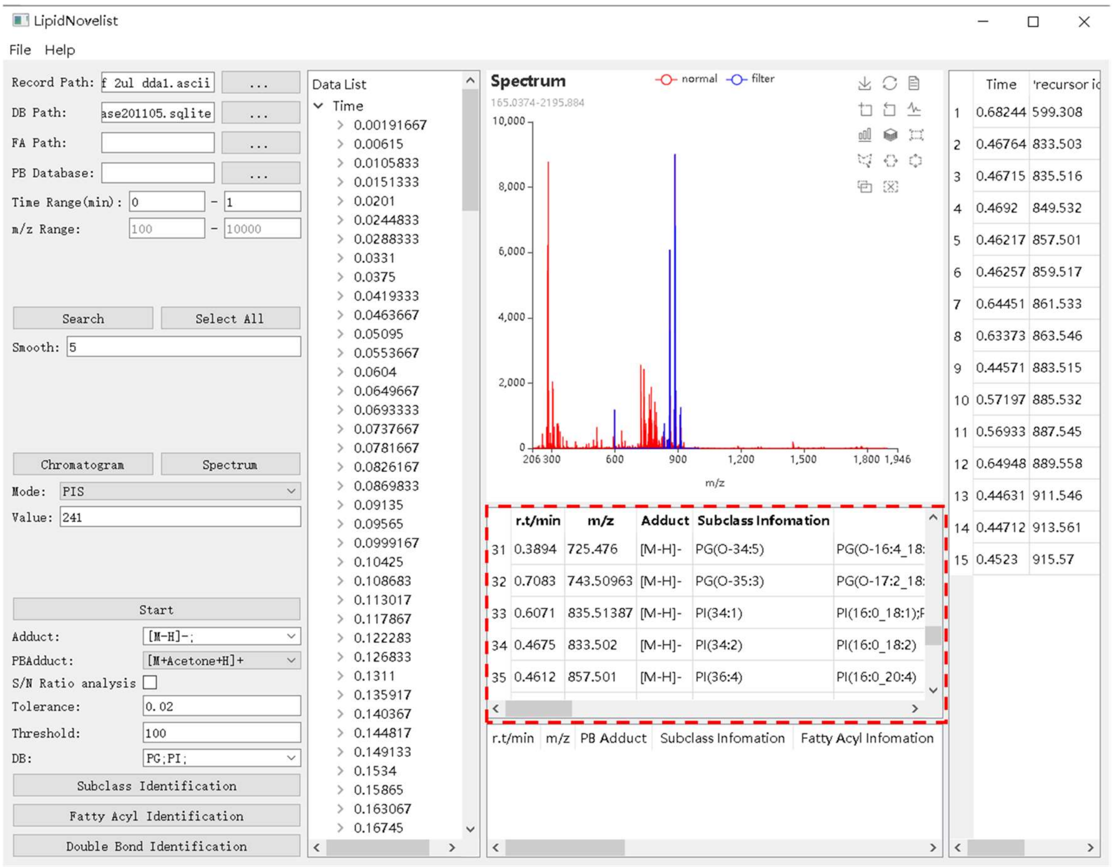

Supplementary Fig. 20. Show lipid identification results at subclass level, chain composition level and *sn*-position level.

For lipid identification at the C=C location level, data from the Paterno-Buchi reaction coupled LC-MS/MS experiment is necessary, and additional parameters must be filled in. The PB-MS/MS annotation requires two steps in lipid annotation. In the first step, a database containing C=Cs in total fatty acids is required, and the PB adduct instead of the adduct must be selected,

with the threshold, tolerance, and lipid species filled (Supplementary Fig. 21). The excel sheet containing the total fatty acids database should have two columns, "chain" and "omega". In the "chain" column, the total fatty acid chain composition is presented in a "X:Y" type, where X represents the number of carbon in a fatty acyl chain, and Y represents the degree of unsaturation in the chain. Information in the 'omega' columns is identified C=Cs in the 'X:Y' fatty acyl chain, where C=Cs locations in polyunsaturated fatty acyl chains are separated by ',' and different locations are separated by ';'. An example, '8, 11, 14; 11, 14, 17' is in the same line of '20:3', which indicates that C20:3(Δ8, 11, 14) and C20:3(Δ11, 14, 17) are identified in total fatty acids in lipid extracts.

After clicking the 'Double bond identification' button in Supplementary Fig. 21, results containing four filled columns are shown in LipidNovelist. These columns are 'r.t.', 'm/z', 'PB Adduct', and 'Subclass information,' where 'm/z' is the m/z of the precursor. In this step, PB-MS/MS precursors are matched by PB adduct and lipid species.

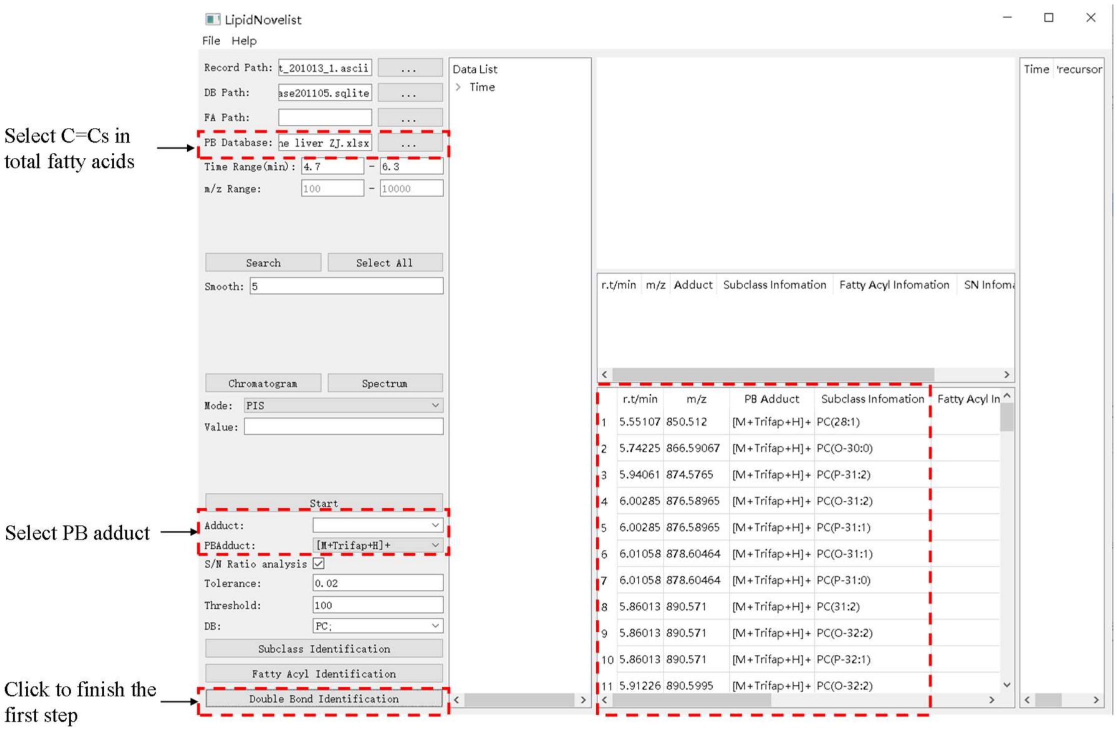

Supplementary Fig. 21. Show process to finish the first step of C=C annotation using data from PB coupled with LC-MS/MS.

Supplementary Fig. 22 displays the second step of PB-MS/MS annotation, which involves the use of an additional excel sheet that contains information on lipid chain composition. The excel sheet consists of two columns, "SUBCLASS" and "FA". The "SUBCLASS" column includes information on lipid subclasses identified using nomenclature such as 'PC(36:1)', while the "FA" column contains information on lipid chain composition within the same subclass, where two more lipid species are separated by ';'.

After processing the excel sheet, the PB-MS/MS annotation table is populated with three columns: "Fatty Acyl Information," "C=C Annotation," and "C18:1 isomeric Ratios." The "Fatty Acyl Information" column lists the fatty acyl chain composition as specified in the excel sheet. The "C=C Annotation" column displays identified lipid C=C locations with diagnostic peaks following each annotation, with different lipid annotations separated by ';'. The "C18:1 isomeric Ratios" column normalizes lipids with C18:1 fatty acyl chains by their C=C diagnostic ions' intensities, and percentage values are displayed following each annotation, and lipid annotations are separated by ';'. An example entry in the table would be "PC(18:0_18:1(Δ9)) 60%; PC(18:0_18:1(Δ11)) 40%" in the same row as "PC(36:1)" in the "Subclass Information" column, "PC 18:0_18:1" in the "Fatty Acyl Information" column, and "PC(18:0_18:1(Δ9)) 678.47, 820.51; PC(18:0_18:1(Δ11)) 706.5, 848.54" in the "C=C Annotation" column. A demonstration of glycerophospholipid annotation at C=C level is available in "Profiling lipid standard at C=C level.mp4" in the tutorial's video section.

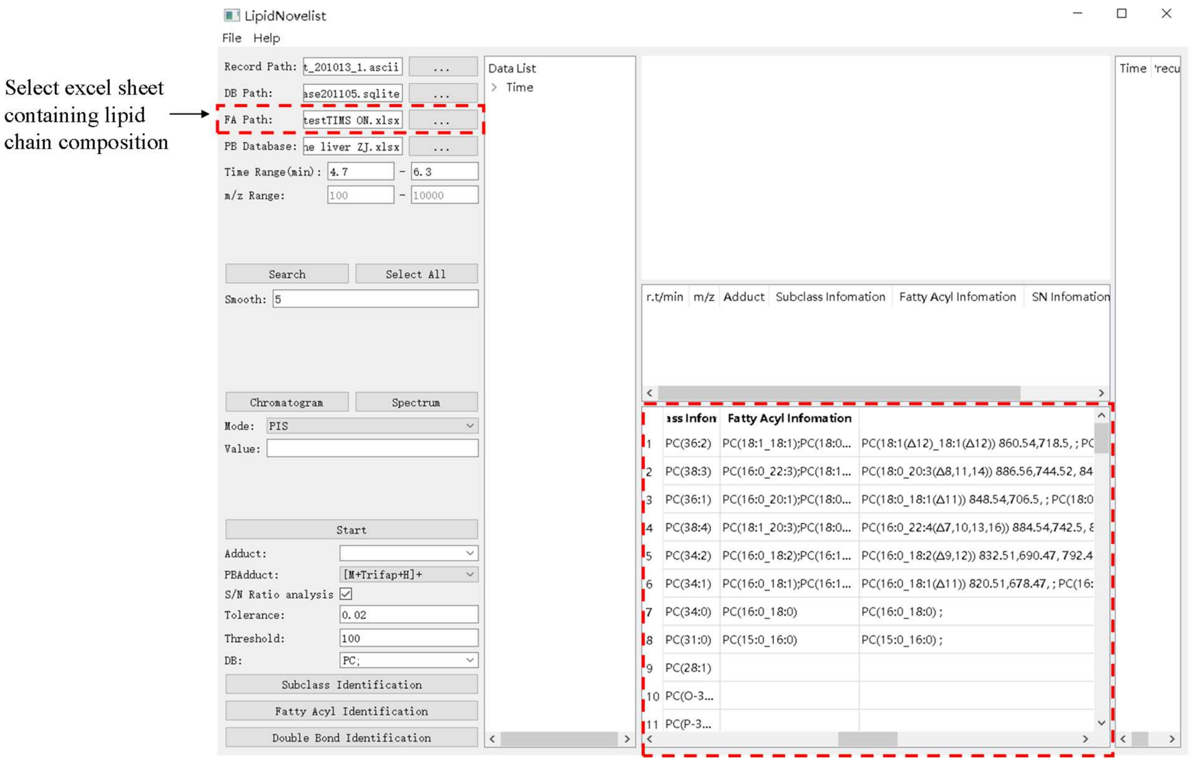

Supplementary Fig. 22. Show process to finish the second step of C=C annotation using data from PB coupled with LC-MS/MS

Another demonstration of total fatty acid annotation at C=C level is available in "fatty acid analysis at C=C level.mp4" in the tutorial's video. During the identification of double bonds in total fatty acids, it is noteworthy that two xlsx files must be imported into both the "FA path" and "PB database". For the previous identification of C=C locations in phospholipids, the xlsx file imported into the "PB database" contained sample-specific total fatty acid information. However, when analyzing total fatty acid 2-acpy PB-MS/MS spectra, the xlsx file imported into the "PB database" only needs to conform to the earlier described format, as its contents will not be utilized during identification.

During the previous identification of C=C positions in phospholipids, the xlsx file imported into the "FA path" contained information regarding lipid chain composition. Given that the subclass information on total fatty acid is equivalent to that of chain composition, the "SUBCLASS" and "FA" columns within the xlsx file should be the same. For instance, if the

"SUBCLASS" column indicates "FA (18:1)", then the "FA" column should likewise indicate "FA (18:1)". When users are identifying C=Cs in total fatty acids via 2-acpy PB-MS/MS spectra, the xlsx file to be imported into the FA path can refer directly to the "total FA_test_modified.xlsx" file. This xlsx file are provided in the "LipidNovelist software & example file/example file/FA path" subfolder of our software package.

Chapter 5 Visualization

To validate results of annotation, or visualize the MS/MS spectra, users can achieve this by selecting a row in the table of identified results or in the subdirectory of the "Raw Data List" section and clicking the right mouse button, as illustrated in Supplementary Fig. 23.

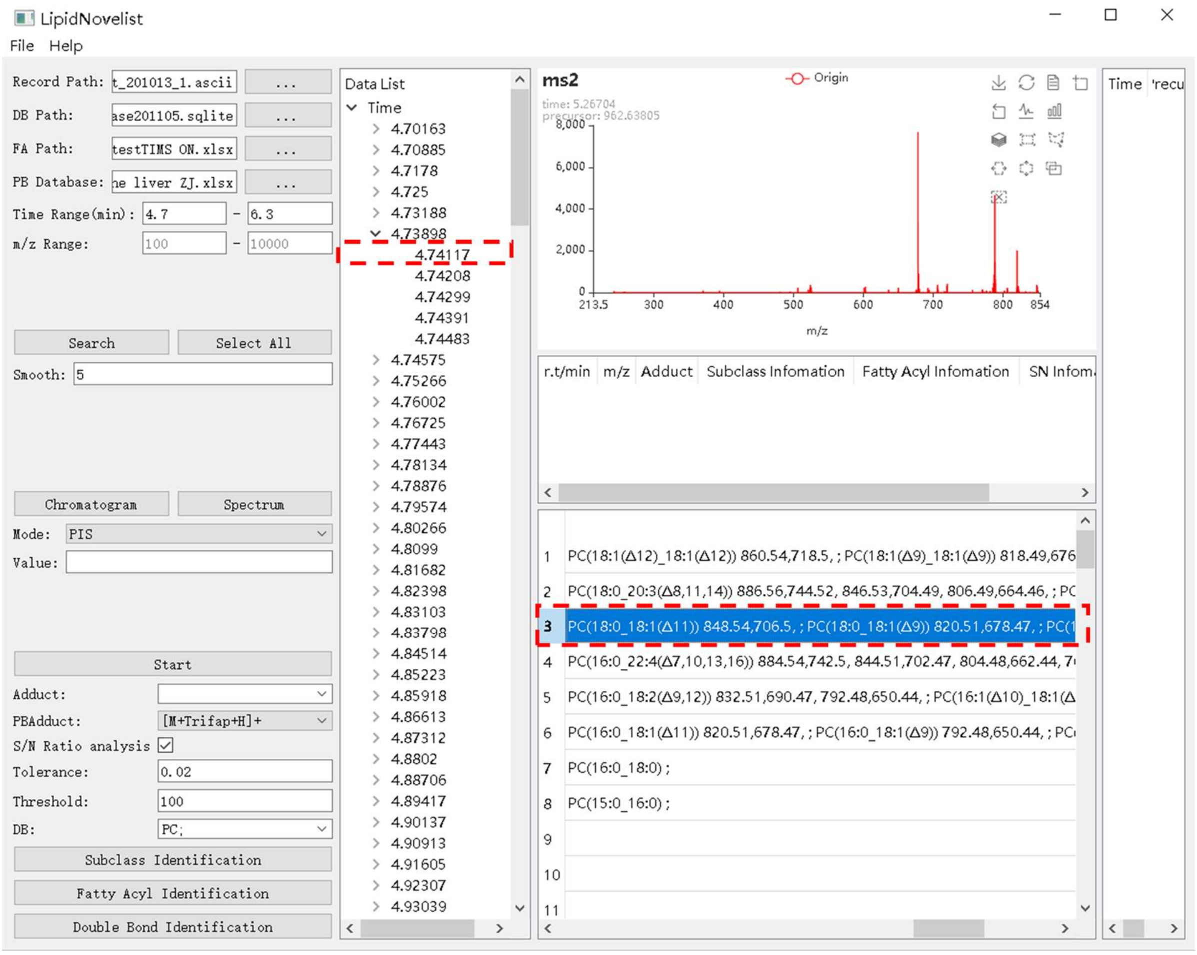

Supplementary Fig. 23. Click the right mouse button and click show to show a spectrum

Overview

LipidNovelist is a python-based GUI software that provides lipid annotation capabilities at the subclass, chain composition, C=C location, and sn location levels. It is the first software to offer near-complete lipid structure annotation. At present, LipidNovelist is designed to read data in .ascii format from MS instruments in Bruker and will soon be able to read data in .mzML format for lipid annotation using data from various MS instruments. We anticipate that LipidNovelist will prove to be a valuable tool for lipid structure annotation and data interpretation in the foreseeable future.

Supplementary Note 2. LipidNovelist Extension - Tutorial

The LipidNovelist Extension is a software tool developed using Python language for performing the relative quantification (I/I_{IS}) of lipids at the sum composition level using liquid chromatography-mass spectrometry (LC-MS) data. The program requires users to import MS^1 spectrum data in .xlsx format and provide information regarding lipid classes, adduct ions, and internal standard m/z for analysis. LipidNovelist Extension performs type I isotope correction on the mass spectrometry data and conducts relative quantification (I/I_{IS}) of lipids at the sum composition level.

The current version of LipidNovelist Extension, including example data and tutorial videos, is available to users for ease of use, and can be found at the following link:

https://doi.org/10.6084/m9.figshare.22297771

A screenshot of the LipidNovelist Extension GUI, as shown in Supplementary Fig. 24, can be divided into two sections: the left section is used for data import and parameter selection, while the right section displays the results of Lipids relative quantification (I/I_{IS}).

In the left section of the GUI, a .sqlite format database sourced from LIPID MAPS is required, which can be imported by clicking on the "Read DB" button. A pre-built .sqlite file named "Lipid-database.sqlite" is provided in the main folder of LipidNovelist Extension

By clicking on the “Read data" button, users can select and read data in the xlsx. format. The excel sheet consists of two columns, "mz" and "inten", which contain mass-to-charge ratio (*m/z*) and intensity information, respectively.

After importing mass spectrum data and .sqlite format database, users must fill in several parameters to obtain successful lipid identification, including the lipid species to be analyzed, the adduct of lipids, the *m/z* of internal standard (IS), and mass tolerance to match peaks with the theoretical *m/z*. Once these parameters are filled in, the user can analyze the spectra to obtain relative quantification results at the sum composition level by clicking the 'Run' button.

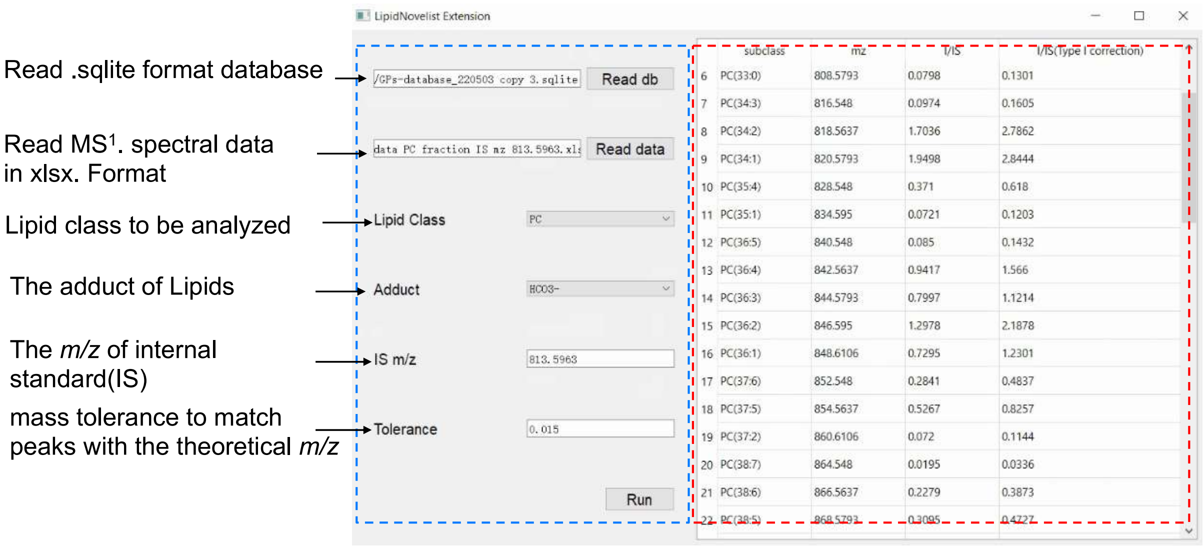

Supplementary Fig. 24. Screenshot of LipidNovelist.

The LipidNovelist Extension displays the results in a table with four filled columns: 'subclass', 'mz', 'I/IS', and 'I/IS(type I correction)'. The 'I/IS' column displays the relative quantification results of lipids without type I correction, while the 'I/IS(type I correction)' column shows the results after type I correction. Typically, relative quantitative results after type I correction are used. For example, an entry in the table may include "PC(36:1)" in the "Subclass" column, "848.6106" in the "mz" column, "0.7295" in the "I/IS" column, and "1.20301" in the "IS(type I correction)" column.

A video tutorial titled "Relative Quantification of Lipids at the Sum Composition Level via LipidNovelist Extension.mp4" provides a demonstration of the relative quantification of lipids at the sum composition level.

Supplementary Methods.

Materials. High-performance LC (HPLC) grade acetonitrile (ACN), iso-propanol (IPA), methanol (MeOH), and water were purchased from Fisher Scientific (Ottawa, ON, Canada). Methyl tert-butyl ether (MTBE), formic acid(HCOOH), ammonium formate, ammonium bicarbonate (NH_4HCO_3) , 2-acetylpiridine (2-acpy) and 2′,4′,6′-trifluoroacetophenone (triFAP) were purchased from Sigma Aldrich (St. Louis, MO, USA). Lipid standards, bovine liver polar extracts and SPLASH Lipidomix were purchased from Avanti Polar Lipids (Alabaster, AL, USA).

Cell cultivation. RAW 264.7 macrophages (American Type Culture Collection (ATCC); Manassas, VA, USA) were cultured in Roswell Park Memorial Institute (RPMI) 1640 Medium with 10% fetal bovine serum and 1% Penicillin-Streptomycin solution and collected by centrifugation. The cell pellets were washed, frozen in liquid nitrogen and stored at −80 °C. For direct inhibition of enzyme activity, RAW 264.7 cells was treated for 72 hours with 60 μM FADS2 inhibitor (SC26196, purchased from Sigma-Aldrich, St Louis, MO, USA) and 2.5 μM SCD -1 inhibitor (CAY10566, purchased from Sigma-Aldrich, St Louis, MO, USA), while the control group was treated with DMSO.

Lipid extraction. A total of 2 million cells were used for lipid extraction. In brief, MTBE (5 mL), MeOH (1.5 mL) and water (1.25 mL) were added to the cell tube(~ 2 million cells). The mixture was then vortexed for 5 min. To separate the organic and aqueous phases, the mixture was centrifuged at 10,000 × g for 10 min. The upper phase was collected. The lower phase was re-extracted with 2 mL of upper phase of system MTBE/MeOH/H_2O (10:3:2.5, v/v/v). Finally, the combined organic phases were collected and dried under N_2 flow for further use. To extract lipids from Bladder cancer tissue, we first homogenized 50 mg of tissue in methanol, added

100uL SPLASH Lipidomix, and followed the extraction protocol described above. All extracts were stored at −20 °C before analysis.

Total fatty acid analysis. The extracted lipids were saponified in 500 μL ACN:15% KOH (50/50, v/v) at 60 °C for 60 min. The solution was acidified with 1M HCl (1 mL). The hydrolyzed total fatty acid were extracted twice with 1.5 mL isooctane each time. The organic layer was collected, dried under nitrogen, and redissolved in an aliquot of 500 μL MeOH for further derivatization. The 2-acpy PB derivatization was performed using a home-made flow microreactor. Total FA extracts and 10 mM 2-acpy were dissolved in 200 μL ACN. The solution was injected into the flow microreactor for 20 s' UV-irradiation (~254 nm). About 200 μL reaction solution was collected; the excess reagent was washed by 600 μL HCl solution (10 mM). The PB derivatized sample was extracted twice by 600 μL isooctane and dried under nitrogen flow. The PB derivatized sample was resuspended in 200 μL MeOH before subsequent RPLC-MS/MS analyses. Reversed phase (RP) LC-MS/MS analyses were conducted on a Waters Acquity UPLC I-Class system (Waters, Milford, MA,USA) hyphenated with a hybrid trapped ion mobility-quadrupole time-of-flight mass spectrometer (timsTOF, Bruker Daltonics, Bremen, Germany). A CORTECS UPLC C18 column (150 mm × 2.1 mm, 1.6 μm, Waters, Milford, MA, USA) was used for separation. The mobile phase A contained H2O:ACN (40:60, v/v, added with 10 mM ammonium formate) and mobile phase B contained IPA:ACN (40:60, v/v, added with 0.1% HCOOH). The flow rate was set at 0.5 mL/min. Oven temperature was set at 60 °C. The chromatographic gradient was as follows: 30% B at 0-0.4 min, 30-45% B at 0.4-0.9 min, 45-52% B at 0.9-1.1 min, 52-58% B at 1.1-1.8 min, 58-66% B at 1.8-2.5 min, 66-70% B at 2.5-3.1 min, 70-75% B at 3.1-4 min, 75-97% B at 4-4.5 min, 97% B at 4.5-6 min, 30% B at 6.1-7 min. The MS parameters were optimized as follows: capillary

voltage, 4500 V; end plate voltage, 500 V; nebulizer, 0.3 Bar; dry gas, 8 L/min; dry temperature, 210 °C; CID energy for MS/MS, 18-25 eV.

TMSD methylation. For PG and PI analysis, a modified TMSD methylation method was employed. Briefly, a solution of TMSD (2.5 M) in hexane (50 µL) was added to the lipid extracts dissolved in methanol (150 µL) to obtain yellow-colored solutions. After vortexing for 30 s, methylation was performed at room temperature for 20 min. Addition of formic acid (5 µL) quenched the methylation and afforded colorless samples, which were then subjected to HILIC-TIMS-MS/MS analysis.

HILIC Separation Conditions. A Waters ACQUITY UPLC I-Class system (Waters, Milford, MA,USA) was used to separate polar lipids on a CORTECS UPLC HILIC column (150 mm × 2.1 mm, 1.6 µm, Waters, Milford, MA, USA). using the following parameters: flow rate 0.5 mL/min, oven temperature 30 °C, mobile phase gradient: 10-15% A at 0-2.4 min, 15-20 % A at 2.4-3.2 min, 20% A at 3.2-5 min, 10% A at 5.1-6 min. Mobile phases A and B were 10 mM NH4HCO_3 aqueous solution and ACN. For methylated phospholipids(^{Me}PL), separation was performed on ACQUITY UPLC BEH Amide Column (150 mm × 2.1 mm, 1.7 µm, Waters, Milford, MA, USA). Mobile phases and flow rate were the same as above. The chromatographic gradient was as follows: 1-2% A at 0-1 min, 2-5% A at 1-2 min, 5-8% A at 2-3 min, 8-10% A at 3-4 min, 10-20% A at 4-4.2 min, 20% A at 4.2-7.0 min, 1% A at 7.1-8 min. The injection volume was 5 µL and each sample was injected three times.

RPLC-MS/MS for TG analysis. RPLC-MS/MS analyses were conducted on a Waters Acquity UPLC I-Class system (Waters, Milford, MA,USA) hyphenated with a hybrid trapped ion mobility-quadrupole time-of-flight mass spectrometer (timsTOF, Bruker Daltonics, Bremen, Germany). A CORTECS UPLC C18 column (150 mm × 2.1 mm, 1.6 µm, Waters, Milford, MA, USA) was used

for separation. The mobile phase A contained H_2O:ACN (40:60, v/v, added with 10 mM ammonium formate) and mobile phase B contained IPA:ACN (90:10, v/v, added with 0.1% HCOOH). The flow rate was set at 0.3 mL/min. Oven temperature was set at 60 °C. The chromatographic gradient was as follows: 70-99% B at 0-7.0 min, 99% B at 7.0-7.5 min, 70% B at 7.6-10 min. The MS parameters were optimized as follows: capillary voltage, 4500 V; end plate voltage, 500 V; nebulizer, 3 Bar; dry gas, 8 L/min; dry temperature, 210 °C; CID energy for MS/MS, 40 eV.

Supplementary References

1. Lerner R*, et al.* Four-dimensional trapped ion mobility spectrometry lipidomics for high throughput clinical profiling of human blood samples. *Nat. Commun.* **14**, 937 (2023).

2. Leaptrot KL, May JC, Dodds JN, McLean JA. Ion mobility conformational lipid atlas for high confidence lipidomics. *Nat. Commun.* **10**, 985 (2019).
