# Supplementary material for: Deep-profiling of phospholipidome via rapid orthogonal separations and isomer-resolved mass spectrometry
Source: Nat Commun. 2023 Jul 17;14:4263. doi: 10.1038/s41467-023-40046-x (PMC10352238; doi:10.1038/s41467-023-40046-x)
Supplement: Supplementary file 2 — Description of Additional Supplementary Files [file 41467_2023_40046_MOESM2_ESM.pdf]

Description of Additional Supplementary Files

File Name: Supplementary Data 1
Description: Identification of phospholipids at the chain level in bovine liver by HILIC-TIMS-MS^2 CID and HILIC-MS^2 CID (Without TIMS separation).

File Name: Supplementary Data 2
Description: Relative compositions (%) of C=C location isomers in 17 groups of total FAs in bovine liver.

File Name: Supplementary Data 3
Description: Identification of phospholipids at the C=C location level in bovine liver by HILIC-TIMS-PB-MS^2 CID.

File Name: Supplementary Data 4
Description: Profiling of phospholipids at the chain and the C=C location level in RAW 264.7 macrophages.

File Name: Supplementary Data 5
Description: Alterations of TG, CE, GPL, and SM at the sum composition level. SPLASH Lipidomix was used as the internal standard for relative quantitation.
