# Supplementary material for: Deep-profiling of phospholipidome via rapid orthogonal separations and isomer-resolved mass spectrometry
Source: Nat Commun. 2023 Jul 17;14:4263. doi: 10.1038/s41467-023-40046-x (PMC10352238; doi:10.1038/s41467-023-40046-x)
Supplement: Supplementary file 8 — Reporting Summary [file 41467_2023_40046_MOESM8_ESM.pdf]

nature portfolio

Corresponding author(s): Yu Xia
Last updated by author(s): Jun 21, 2023

Reporting Summary

Nature Portfolio wishes to improve the reproducibility of the work that we publish. This form provides structure for consistency and transparency in reporting. For further information on Nature Portfolio policies, see our Editorial Policies and the Editorial Policy Checklist.

Statistics

For all statistical analyses, confirm that the following items are present in the figure legend, table legend, main text, or Methods section.

n/a	Confirmed	
☐	☒	The exact sample size (*n*) for each experimental group/condition, given as a discrete number and unit of measurement
☐	☒	A statement on whether measurements were taken from distinct samples or whether the same sample was measured repeatedly
☐	☒	The statistical test(s) used AND whether they are one- or two-sided<br>*Only common tests should be described solely by name; describe more complex techniques in the Methods section.*
☒	☐	A description of all covariates tested
☒	☐	A description of any assumptions or corrections, such as tests of normality and adjustment for multiple comparisons
☐	☒	A full description of the statistical parameters including central tendency (e.g. means) or other basic estimates (e.g. regression coefficient) AND variation (e.g. standard deviation) or associated estimates of uncertainty (e.g. confidence intervals)
☐	☒	For null hypothesis testing, the test statistic (e.g. *F*, *t*, *r*) with confidence intervals, effect sizes, degrees of freedom and *P* value noted<br>*Give P values as exact values whenever suitable.*
☒	☐	For Bayesian analysis, information on the choice of priors and Markov chain Monte Carlo settings
☐	☒	For hierarchical and complex designs, identification of the appropriate level for tests and full reporting of outcomes
☒	☐	Estimates of effect sizes (e.g. Cohen's *d*, Pearson's *r*), indicating how they were calculated

Our web collection on statistics for biologists contains articles on many of the points above.

Software and code

Policy information about availability of computer code

Data collection	MS raw data were recorded with Bruker otofControl version 5.0
Data analysis	Data Analysis 5.0 software from Bruker, LipidNovelist&LipidNovelist Extension(a home-made software). The most up-to-date version of LipidNovelist & LipidNovelist Extension, along with instructional videos and example data to facilitate its utilization, can be freely accessed at https://doi.org/10.6084/m9.figshare.22297771The most up-to-date version of LipidNovelist & LipidNovelist Extension, along with instructional videos and example data to facilitate its utilization, can be freely accessed at https://doi.org/10.6084/m9.figshare.22297771

For manuscripts utilizing custom algorithms or software that are central to the research but not yet described in published literature, software must be made available to editors and reviewers. We strongly encourage code deposition in a community repository (e.g. GitHub). See the Nature Portfolio guidelines for submitting code & software for further information.

Data

Policy information about availability of data

All manuscripts must include a data availability statement. This statement should provide the following information, where applicable:
- Accession codes, unique identifiers, or web links for publicly available datasets
- A description of any restrictions on data availability
- For clinical datasets or third party data, please ensure that the statement adheres to our policy

All data necessary to support the conclusions are available in the manuscript or supplementary information. The raw MS data are available from Figshare.

(https://doi.org/10.6084/m9.figshare.21946100). Source data are provided with this paper.

Human research participants

Policy information about studies involving human research participants and Sex and Gender in Research.

Reporting on sex and gender	One female and five males were included in the study. However, due to the small sample size, we were unable to conduct sex- and gender-based analyses.
Population characteristics	The human bladder cancer tissue samples were obtained from a cohort of six Asian individuals, comprising one female and five males. The age range of the participants was 47 to 83 years.
Recruitment	Not relevant. Samples were obtained from specimen bank of the hospital.
Ethics oversight	The study protocol was approved by the Ethical Review Board of Tsinghua University.

Note that full information on the approval of the study protocol must also be provided in the manuscript.

Field-specific reporting

Please select the one below that is the best fit for your research. If you are not sure, read the appropriate sections before making your selection.

☒ Life sciences ☐ Behavioural & social sciences ☐ Ecological, evolutionary & environmental sciences

For a reference copy of the document with all sections, see nature.com/documents/nr-reporting-summary-flat.pdf

Life sciences study design

All studies must disclose on these points even when the disclosure is negative.

Sample size	The study design for RAW 264.7 cell line did not consider sex as a factor. These samples were solely used to illustrate the application of the developed lipidomic approach. A sample size of 6 was chosen for the experiments to achieve meaningful conclusions from the data, and this sample size also demonstrated the reproducibility of the lipid analysis results.
Data exclusions	No data were excluded.
Replication	To ensure the reliability and robustness of our experimental results, we performed analysis on six biological replicates, each cell sample accompanied by three independent experiments.
Randomization	For enzyme activity inhibition experiment, RAW 264.7cells were grouped to Control/CAY10566/SC26196 groups according to whether the drug was used. No further grouping was applied.
Blinding	Blinding is not applicable to the technical benchmarks described.

Reporting for specific materials, systems and methods

We require information from authors about some types of materials, experimental systems and methods used in many studies. Here, indicate whether each material, system or method listed is relevant to your study. If you are not sure if a list item applies to your research, read the appropriate section before selecting a response.

Materials & experimental systems

n/a	Involved in the study	
☒	☐	Antibodies
☐	☒	Eukaryotic cell lines
☒	☐	Palaeontology and archaeology
☒	☐	Animals and other organisms
☒	☐	Clinical data
☒	☐	Dual use research of concern

Methods

n/a	Involved in the study	
☒	☐	ChIP-seq
☒	☐	Flow cytometry
☒	☐	MRI-based neuroimaging

Eukaryotic cell lines

Policy information about cell lines and Sex and Gender in Research

Cell line source(s)	RAW 264.7 cell line was obtained from American Type Culture Collection (Manassas, VA, USA)
Authentication	Cell lines authenticated by short tandem repeat (STR) testing.
Mycoplasma contamination	We confirm that all cell lines tested are negative for mycoplasma contamination.
Commonly misidentified lines (See ICLAC register)	No commonly misidentified lines used.
